# Adapting and Evaluating a Brief Advice Tobacco Cessation Intervention in High-reach, Low-resource Settings in India: Protocol for a Cluster Randomized Controlled Trial

**DOI:** 10.2196/57236

**Published:** 2024-09-03

**Authors:** Sitara L Mahtani, Kasisomayajula Viswanath, Himanshu A Gupte, Gauri Mandal, Dinesh Jagiasi, Ratandeep Chawla, Marina D'Costa, Ziming Xuan, Sara Minsky, Shoba Ramanadhan

**Affiliations:** 1 Dana-Farber Cancer Institute Boston, MA United States; 2 Harvard T.H. Chan School of Public Health Boston, MA United States; 3 Narotam Sekhsaria Foundation Mumbai India; 4 Salaam Bombay Foundation Mumbai India; 5 Boston University School of Public Health Boston, MA United States

**Keywords:** tobacco use Cessation, India, resource-limited settings, task-shifting, counseling, nonprofit organizations, dental health services, tuberculosis, social media, mobile app

## Abstract

**Background:**

About 1.35 million deaths annually are attributed to tobacco use in India. The main challenge, given the magnitude of tobacco use and limited resources, is delivering cessation support at scale, low cost, and through a coordinated cross-system effort; one such example being brief advice interventions. However, highly credentialed staff to identify and counsel tobacco users are scarce. Task-shifting is an important opportunity for scaling these interventions.

**Objective:**

The LifeFirst SWASTH (Supporting Wellbeing among Adults by Stopping Tobacco Habit) program—adapted from the LifeFirst program (developed by the Narotam Sekhsaria Foundation, Mumbai, India)—is a tobacco cessation program focusing on lower-socioeconomic status patients in Mumbai receiving private health care. This parallel-arm, cluster randomized controlled trial investigates whether the LifeFirst SWASTH program increases tobacco cessation rates in low-resource, high-reach health care settings in Mumbai.

**Methods:**

This study will target tuberculosis-specific nongovernmental organizations (NGOs), dental clinics, and NGOs implementing general health programs serving lower-socioeconomic status patients. Intervention arm patients will receive a pamphlet explaining tobacco’s harmful effects. Practitioners will be trained to deliver brief cessation advice, and interested patients will be referred to a Narotam Sekhsaria Foundation counselor for free telephone counseling for 6 months. Control arm patients will receive the same pamphlet but not brief advice or counseling. Practitioners will have a customized mobile app to facilitate intervention delivery. Practitioners will also have access to a peer network through WhatsApp. The primary outcome is a 30-day point prevalence abstinence from tobacco. Secondary outcomes for patients and practitioners relate to intervention implementation.

**Results:**

The study was funded in June 2020. Due to the COVID-19 pandemic, the study experienced some delays, and practitioner recruitment commenced in November 2023. As of July 2024, all practitioners have been recruited, and practitioner recruitment and training are complete. Furthermore, 36% (1687/4688) of patients have been recruited.

**Conclusions:**

It is hypothesized that those patients who participated in the LifeFirst SWASTH program will be more likely to have been abstinent from tobacco for 30 consecutive days by the end of 6 months or at least decreased their tobacco use. LifeFirst SWASTH, if found to be effective in terms of cessation outcomes and implementation, has the potential to be scaled to other settings in India and other low- and middle-income countries. The study will be conducted in low-resource settings and will reach many patients, which will increase the impact if scaled. It will use task-shifting and an app that can be tailored to different settings, also enabling scalability. Findings will build the literature for translating evidence-based interventions from high-income countries to low- and middle-income countries and from high- to low-resource settings.

**Trial Registration:**

ClinicalTrials.gov NCT05234983; https://clinicaltrials.gov/study/NCT05234983

**International Registered Report Identifier (IRRID):**

DERR1-10.2196/57236

## Introduction

### Tobacco Burden and Control in India

About 1.35 million deaths are attributed to tobacco use in India each year [1]. In 2017 [2], there were roughly 267 million tobacco users including 199 million smokeless tobacco users, 100 million smokers, and 32 million dual users. Per the nationally representative 2016-2017 Global Adult Tobacco Survey (GATS-2), tobacco use is higher among men than women (42.4% or 32,436/76,500 men vs 14.2% or 10,863/76,500 women). Cessation is of interest to tobacco users in India; 38.5% (29,453/76,500) of smokers and 33.2% (about 25,398/76,500) of smokeless tobacco users reported a quit attempt in the year preceding the survey [2]. Although India is a signatory to the World Health Organization (WHO) Framework Convention on Tobacco Control and launched the National Tobacco Control Program in 2007 [3], the program was hampered by limited resources and inconsistent enforcement across states and regions [4]. The main challenge in India, given the magnitude of tobacco use and limited resources, is in delivering cessation supports at scale, at low cost, and through a coordinated effort among those engaged in tobacco cessation: nongovernmental organizations (NGOs) and clinics [5].

### Barriers to Scaling Up Evidence-Based Tobacco Cessation in India

Low-resource settings require tobacco cessation evidence-based interventions (EBIs) that can be delivered, sustained, and scaled up at low cost [6-8]. However, tobacco cessation EBIs typically include pharmacotherapy, a resource still beyond the reach of most tobacco users and many health care systems in India [9], even though a limited number of pharmacotherapeutic treatments were recently added to the Indian Ministry of Health and Family Welfare’s List of Essential Medicines [10]. Also, most tobacco cessation EBIs focus solely on cigarette smoking, which is a mismatch for India given the high rates of smokeless and dual tobacco use [11], and the fact that the smokeless tobacco market in India is the largest in the world [12]. Additionally, EBIs are more likely to be scaled up in low- and middle-income countries (LMICs) if they are simple and inexpensive and can be delivered via alternative channels, such as NGOs or small private practices, rather than relying entirely on government infrastructure and highly skilled personnel [13]. Finally, there is an overall lack of effective interventions to support health care practitioners in implementing evidence-based practices across various health topics in LMICs [14].

### Brief Advice Interventions

One type of low-cost, scalable cessation support is brief advice interventions, or short engagement by health care providers to screen for tobacco use, offer brief cessation advice, and refer users to treatment resources [15]. Brief advice interventions are effective for both smoked and smokeless tobacco [16,17]. However, according to the GATS-2 in India, only 49.6% (37,332/76,500), of smokers and 31.7% (24,251/76,500) of smokeless tobacco users were advised to quit by a health care provider [2], highlighting a potential role for brief advice interventions [18].

### Task-Shifting: Benefits and Training Requirements

The use of diverse health professionals (rather than only highly credentialed individuals) is an important opportunity for brief advice interventions [9]. India meets only 25% of the WHO benchmark for health care staffing, and the available educational facilities are insufficient to grow the workforce to needed levels [19]. To reach the needed staffing levels, the number of physicians would need to increase by 151%, but the current annual growth rate is 10%-14% [20]. Task-shifting is a strategy that emphasizes moving tasks from highly credentialed workers to less credentialed (but more readily available) workers and can promote access to preventive services and health. The task-shifting model has been effectively used to deliver mental health EBIs in LMICs, showing that health care providers with little or no prior mental health training, and often little formal education, can deliver EBIs effectively with positive clinical outcomes [21]. An important consideration for task-shifting models is the changes needed to adapt practitioner training to meet the needs of practitioners who have fewer formal credentials. For example, an EBI for autism spectrum disorder was adapted from the United Kingdom to sites in India and Pakistan. The use of nonspecialists to deliver the program necessitated a change from a 2-day training for therapists to a 4-week training for nonspecialists and new referral patterns and measures for nonspecialist competencies [22]. Another study trained community health workers (CHWs) in Tamil Nadu, India, to screen, examine, refer to further care, and follow up with patients with diabetes with the support of a tablet-based app. The CHWs were successful in their efforts compared to usual care, further indicating the use of task-shifting [23] The specific question addressed by this study is whether brief advice interventions can be delivered by a diverse range of health professionals instead of highly trained counselors.

### Delivering Sufficient Training Dose Using Technology

Practitioner training is a critical driver of program success [24,25] and can increase practitioners’ knowledge, skills, motivation, and ability to implement EBIs [25-30]. Successful training requires spending enough time (achieving sufficient dose) and offering supports (such as ongoing technical assistance), connections, and resources, a challenge in low-resource settings [29]. Communication technologies, such as mobile phones, offer a tremendous opportunity and have been used with frontline health workers to increase knowledge and skills, deliver learning materials and reminders, improve guideline adherence, increase patient follow-up rates, increase worker credibility in the community, and support knowledge generation [31-34]

### The Current Intervention

Narotam Sekhsaria Foundation (NSF) is an organization that has been working on tobacco cessation programs at various sites in the Mumbai Metropolitan Region (MMR) in India for more than 10 years. The NSF team developed and implemented LifeFirst, an evidence-informed tobacco cessation program involving cessation service provision in multiple settings including hospitals, workplaces, and schools, and conducting training for health care providers. It is based on EBIs developed in North America and Europe using the 5A’s framework [35-38]. The 5A’s framework (Ask, Advise, Assess, Assist, and Arrange) is a tool to help practitioners identify tobacco users, advise quitting, assess their readiness to quit, help them develop a plan, and arrange a follow-up visit or consultation [39]. The training builds skills and motivation to offer brief advice routinely and has two tiers: (1) a sensitizing version that orients practitioners to the need for tobacco control and builds skills for brief advice (Level 1) and (2) an intensive version to support practitioners in tobacco cessation counseling (Level 2).

The NSF team recently delivered the Level 1 training to 2000 practitioners. Before this, most participants had not received training to screen clients for tobacco use (83% or 1660/2000) or to provide cessation counseling (86% or 1720/2000). After receiving the training, participants reported increased knowledge to support patients and improved self-efficacy for counseling. Additionally, the team recently delivered the Level 2 training to 600 diverse health care providers from private and public hospitals, private practices, public health departments, and NGOs in Mumbai. Recently, NSF identified the potential for NGOs working on tuberculosis (TB) and treatment to deliver tobacco cessation, along with a need for less intensive interventions, setting the stage to test a brief intervention that requires few resources to implement [40].

The LifeFirst program has been adapted to develop LifeFirst Supporting Wellbeing among Adults by Stopping Tobacco Habit (SWASTH), which focuses on lower-socioeconomic status (SES) communities in Mumbai who receive health care through private dental practices and NGOs, including those working with TB patients (ClinicalTrials.gov NCT05234983). (“Swasth” means “healthy” in Hindi.) It is an EBI that involves training health care practitioners who serve lower-SES communities to ask their patients about tobacco use during health-related encounters, advise users to quit, and refer users willing to receive cessation support to LifeFirst SWASTH for 6 months’ cessation counseling. Counseling will be conducted by trained NSF staff counselors. The practitioners and counselors will use a customized mobile app to facilitate intervention delivery. Practitioners will also have access to a peer network through the social media platform WhatsApp, which will enable them to problem-solve together and enhance their capacity to implement EBIs in their clinics or practices.

### Objectives

Our objectives are as follows:

- Aim 1: adapt the LifeFirst program and pilot-test LifeFirst SWASTH in 3 low-resource, high-reach settings: NGOs implementing general health programs, TB-specific NGOs, and dental clinics.
- Aim 2: conduct a cluster randomized controlled trial (RCT) to assess whether the adapted brief advice program results in increased cessation rates among tobacco users (compared to receiving a pamphlet explaining the health effects of tobacco use) in the aforementioned 3 types of low-resource, high-reach health care settings in Mumbai.
- Aim 3: evaluate the use of communication technologies, such as social media and an app, to support ongoing training and networking among practitioners in the intervention arm.

Our main focus in this paper is on effectiveness outcomes and will report more robustly on our exploratory implementation findings in the future.

### Theoretical Framework

**Figure 1:**
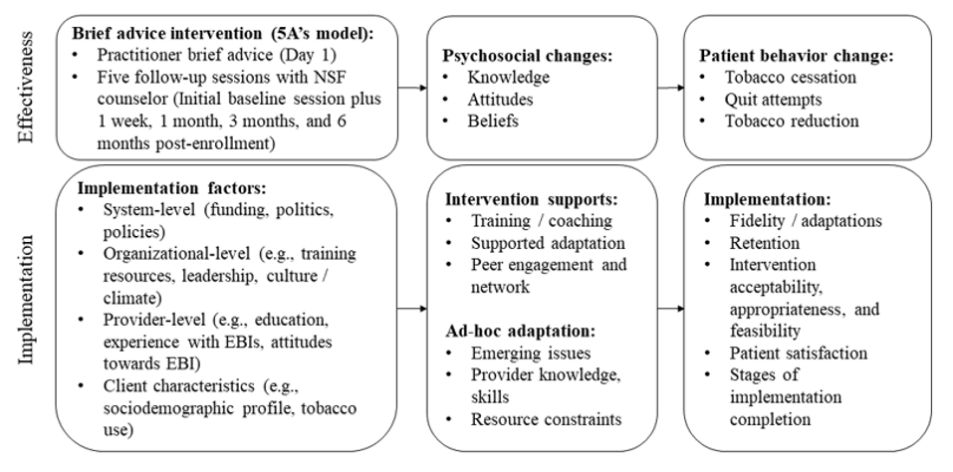
Theoretical framework supporting a hybrid effectiveness and implementation study. EBI: evidence-based intervention; NSF: Narotam Sekhsaria Foundation.

The objective of this study is to test the effect of an adapted EBI, LifeFirst SWASTH, compared to receiving a pamphlet about the health effects of tobacco use, on improving cessation rates among tobacco users in 3 types of low-resource, high-reach health care settings in Mumbai: NGOs implementing general health programs, TB-specific NGOs, and dental clinics. This Hybrid Randomized Control Trial Type 1 study [41] will evaluate program effectiveness and explore the implementation of LifeFirst SWASTH, drawing on the US Public Health Service 5A’s model of tobacco control [42] and the Exploration, Preparation, Implementation Sustainment framework [24]. Figure 1 details the study’s theoretical framework. We hypothesize that 30-day point prevalence abstinence (PPA) will increase, and tobacco use will decrease at 6-month follow-up among patients in the adapted EBI, LifeFirst SWASTH, arm compared to the patients in the control arm.

## Methods

### Design Overview

**Figure 2:**
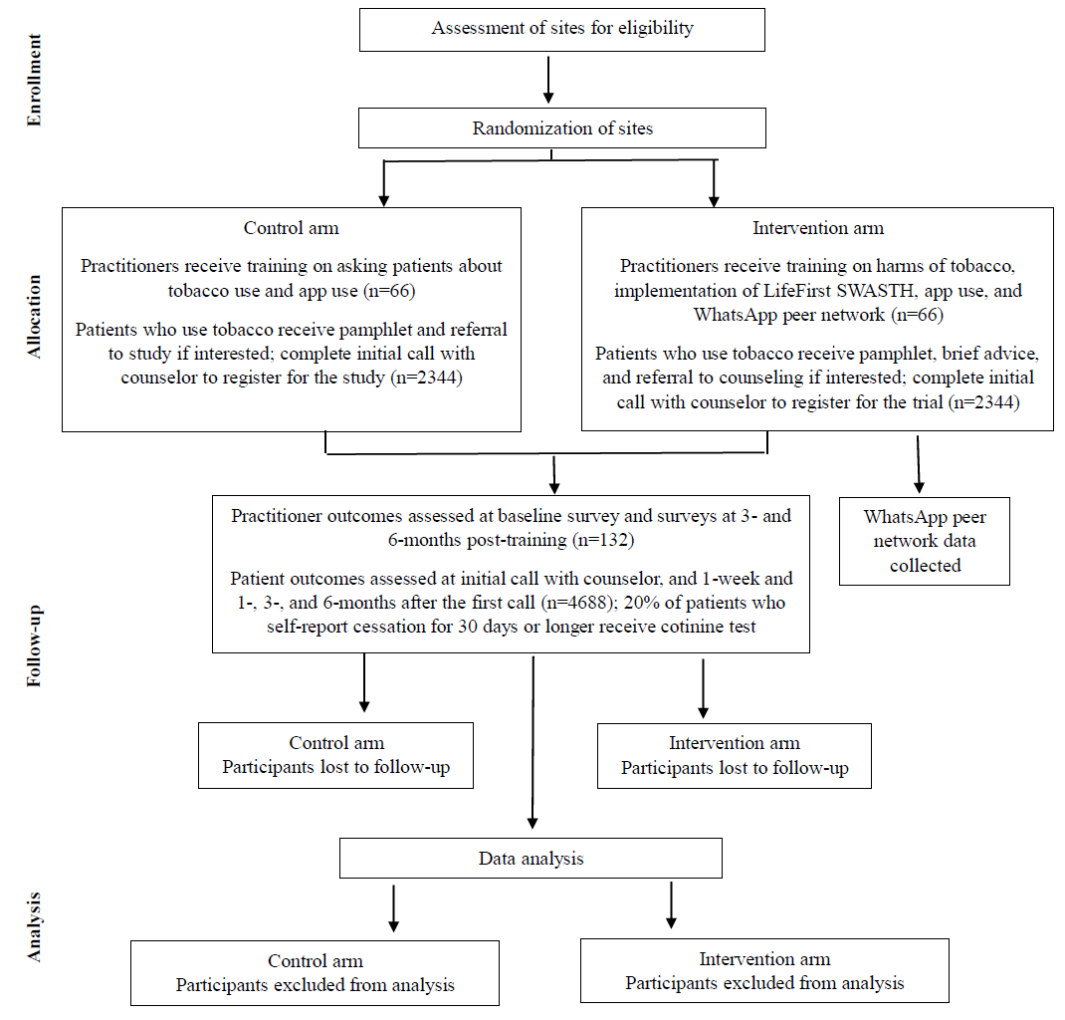
CONSORT (Consolidated Standards of Reporting Trials) flow diagram. LifeFirst SWASTH: LifeFirst Supporting Wellbeing among Adults by Stopping Tobacco Habit.

This study is a parallel-arm cluster RCT. Textbox 1 outlines the eligibility criteria for study sites. Textbox 2 details the participant eligibility criteria for practitioners and patients. Figure 2 provides an overview of the intervention design and flow.

**Site eligibility criteria.**

- Private (separate from the public or government-run health care system) nongovernmental organizations implementing general health programs, tuberculosis nongovernmental organizations, or dental practice
- Serves lower-socioeconomic status patients
- Located in the Mumbai Metropolitan Region
- Has not previously received LifeFirst counseling training (Level 2)
- Is not currently running a tobacco cessation program

**Participant eligibility criteria.**

**Practitioner eligibility (n=132)**

- Is aged 18 years and older
- Is a targeted worker from a targeted setting (an outreach worker in a nongovernmental organization implementing general health programs, a community health worker in a tuberculosis-specific nongovernmental organization, or a dentist in private practice)
- Sees lower-socioeconomic status patients
- Has sufficient language proficiency in English and/or Hindi to attend the training and take surveys, and have sufficient language proficiency in Hindi and/or Marathi to interface with patients
- Has not received prior training in tobacco cessation counseling
- Can recruit 10-60 tobacco users in a 6-month period
- Works in a setting in which brief advice can be added to the workflow
- Delivers care in the Mumbai Metropolitan Region and expects to continue doing so for 1 year after completing the Supporting Wellbeing among Adults by Stopping Tobacco Habit practitioner training
- Can secure institutional signoff to participate and use the program in practice
- Has an Android smartphone

**Patient eligibility (n=4688)**

- Has used smokeless or smoked tobacco even once in the past 30 days
- Is aged 18 years and over
- Speaks Hindi or Marathi
- Attends the practice of an enrolled practitioner
- Has a mobile phone (required for data collection)
- Does not have another member of their household participating in the study
- Is not currently participating in any tobacco cessation program (such as through the National Quitline)

### Recruitment and Retention

The NSF team has rich experience in recruiting practitioners who meet these criteria and is already working with practitioners and leaders in each of the 3 practice settings in Mumbai. The team will leverage these connections to recruit a diverse pool of sites and practitioners. Additionally, all practitioners and patients in both the intervention and control arms will receive nominal monetary incentives for their time and effort. Participating NGOs will also receive nominal monetary incentives at the site level. As private dental practices are run by dentists (who are, in this case, practitioners), they will not receive an additional site-level incentive.

### Randomization

Sites will be randomized to conditions using random number generators in the SAS (version 9.4; SAS Institute). To ensure equivalence across treatment conditions, we will stratify random assignment based on two factors: (1) clinic types (eg, TB-specific NGOs, dental clinics, and NGOs implementing general health programs); (2) size of clinics with respect to the numbers of health care providers and patients. Randomization will be conducted within clinic types and matched by clinics with similar sizes. We will check the balance of treatment assignment on these 2 factors to assess the result of the random assignment. The health care providers and patients will not be blinded to their intervention condition.

A parallel study design will be used [43]. This design is considered the gold standard in assessing unbiased treatment effects given that randomization helps mitigate bias from known and unknown confounders. After randomization, individuals in each arm will stay in their assigned condition for the duration of the study. A cluster parallel RCT has the advantage of preventing inadvertent exposure to intervention or cross-over, thus reducing the risk of contamination [43].

### Intervention Arm

In LifeFirst SWASTH, patients who use tobacco will receive a pamphlet in Hindi about the harmful effects of tobacco. Practitioners will be trained to deliver the brief advice intervention. They will use an app to facilitate ongoing engagement and learning related to the adapted brief advice intervention. The app offers:

(1) a system to document patients’ tobacco use and refer them to LifeFirst SWASTH for counseling; (2) a dashboard allowing them to follow the progress of the patients they have referred; and (3) a repository of training materials and additional resources, for example, support material for promoting cessation, and quiz questions to test their knowledge.

An NSF-moderated group chat in the popular social media channel, WhatsApp, will also be used as a forum to share practitioners’ innovations and successes.

Within 15 days of referral, patients will be contacted by a trained NSF counselor over the telephone, given more details about the study, and asked whether they would like to provide informed consent and participate. The counselors will follow up with the patients for a total period of 6 months, with 4 specific follow-up intervals at 1 week, 1 month, 3 months, and 6 months after the initial detailed counseling call to ask about tobacco use and provide continued support.

### Control Arm

Patients in the control arm will be asked about tobacco use and will receive the same pamphlet as the intervention arm about the harmful effects of tobacco. Patients interested in participating in the study will be referred by practitioners to LifeFirst SWASTH. They will be contacted by NSF data collectors (who are not trained in counseling) over the telephone at the same 4 follow-up intervals as the intervention arm patients but will only be asked about their tobacco use status (this will be presented as NSF assessing the tobacco cessation landscape in MMR). During the RCT, if control patients request counseling, they will be referred to the NSF's regular programmatic counselors and withdrawn from the study. They will subsequently be assigned to the lost-to-follow-up group (due to requesting counseling). After their final follow-up, those control arm patients who have not quit will be offered LifeFirst counseling by NSF staff through the regular counseling program, separate from the study.

Like intervention arm practitioners, practitioners in the control arm will use an app to document patients’ tobacco use and register patients to be followed up by NSF. However, they will not be trained on the harms of tobacco use or advising tobacco-using patients on quitting and referring them to counseling. They will also not be part of a WhatsApp peer network. The training for control arm practitioners will emphasize that all patients will be given the option to access the intervention, including counseling, after their final follow-up with NSF. The control arm practitioners will also be offered the opportunity to receive the training after data collection is complete.

### Primary Outcome: Intervention Effectiveness

The primary outcome is a 30-day PPA from tobacco. Twenty percent of those who self-report tobacco cessation 6 months post recruitment will receive a cotinine test (using a urine sample) to biochemically verify abstinence.

### Secondary Outcomes

#### Patients

The secondary outcomes for patients are quit attempts, reduction in tobacco use, and psychosocial antecedents of behavior change such as knowledge, attitudes, and perceptions regarding the use of smoked or smokeless tobacco. These questions are primarily drawn from the GATS-2 survey, which was standardized, pretested in the field, and validated before being executed throughout India [2]. For the intervention arm patients, the survey includes questions regarding plans to quit and whether they have accessed other supports to do so, which will be tracked over time to assess the patient’s progress and readiness to quit.

#### Practitioners

The secondary outcomes for practitioners are knowledge, appropriateness, feasibility, acceptability, motivation to implement the intervention, and intervention delivery skills. The appropriateness, feasibility, and acceptability questions were adapted from the Intervention Appropriateness Measure, Feasibility of Intervention Measure, and Acceptability of Intervention Measure [44]. The original measures were validated thoroughly by Weiner and colleagues and demonstrated substantive and discriminant content validity [44]. We recognize that the populations with which the original validation studies were conducted are dissimilar from our practitioner groups, and thus will conduct cognitive testing of the items. Some questions, such as those measuring knowledge, are standard questions the NSF team uses for their LifeFirst training.

#### Counselors

The secondary outcomes for counselors will be the appropriateness, feasibility, and acceptability of the intervention. This information will be documented by the NSF team, as the counselors are NSF staff members.

### Data Collection and Analysis or Statistical Considerations

We will first examine the demographic characteristics of the intervention and control arms to verify that randomization has resulted in arms with similar baseline characteristics. Analyses will be based on intent-to-treat. To control for potential confounders identified in bivariate analyses, as well as variables of a priori clinical significance (SES, gender, age), we will regress the 6-month follow-up outcomes on the intervention status with the adjustment of baseline measures. We will use a regression model with a logit link function for binary outcomes (eg, tobacco cessation as our primary outcome), an identity link function for continuous outcomes (eg, number of cigarettes), and a Poisson link function for count outcomes (eg, number of attempts to quit). We will use robust standard error estimates (Generalized Estimating Equations method), adjusting for the clustering of patients among practitioners. We will conduct stratified analyses by clinic types to assess potential effect modification by settings. Independent variables with strong high correlations may result in collinearity. To assess the extent of collinearity, we will assess variance inflation factors and the standard error estimates for the adjusted model.

We will also measure the receipt of intervention using process data from the NSF team and conduct a dose-response analysis of the program impact. We will categorize patients in the intervention arm into 6 subgroups (brief advice, brief advice + intake, brief advice + intake + 1 follow-up, brief advice + intake + 2 follow-ups, etc). The mobile app will capture this information. While the study is not powered to detect differences between the subgroups definitively, we expect to be able to detect trends in response to the level of receipt of intervention, contingent on the distribution of exposure across subgroups.

### Power Analysis and Sample Size

Our sample size estimates were calculated conservatively. Based on the estimates of a risk ratio of 1.75 and the proportion (3.72%) of the control arm who quit smoking (216/5811) in a recent meta-analysis of brief advice interventions [45], we calculated an estimated proportion (6.50%) of the intervention arm who received the brief tobacco intervention and quit tobacco use. We are conducting a cluster RCT with practice settings as the unit of random assignment. Assuming an intraclass correlation of 0.01 and the potential pool of practitioners to be 131, we will need about 36 tobacco-using patients for each practitioner to have a total sample size of 2344 tobacco users per arm, based on a type I error rate of 0.05 and 80% statistical power. Thus, our proposed final sample size is 4688, accounting for 20% attrition among practitioners and 30% among patients. Our effect size estimate is conservative in light of a recent study suggesting a 12.5% quit rate at 6 weeks among smokers receiving behavioral counseling in a pre-post comparison without a control arm receiving standard care in India [9]. Although the 6-month quit rate tends to be lower than the 6-week quit rate after brief advice, we expect our conservative sample size estimate should provide adequate statistical power.

### Informed Consent

During their initial in-person training, study staff will review the consent form with practitioners and obtain written consent. Practitioners will take preliminary verbal consent from patients who are interested and eligible and document it in the app. The counselors and data collectors will be able to view patients who have provided preliminary consent. During the first phone call with the patient, the NSF counselor or data collector will review the participation consent form with the referred patients over the phone and answer any questions. The institutional review board (IRB) has granted us a waiver of written consent for the patients, given that they will be interacting with the counselors and data collectors over the phone. After the 6-month follow-up call, if a patient has been randomly selected for cotinine testing, the counselor or data collector will double-check that they still consent to the test. The NSF team will use data from the app to document who has provided consent. It will be made clear during the consent process that all participants (practitioners and patients) may decide not to participate at any time with no consequences.

### Privacy and Confidentiality

All identifiable data will be securely stored with the NSF team. They will remove the data of any identifiable information and save the deidentified data in password-protected Cloud-based files. Only deidentified data will be used for analysis. Urine specimens collected for the cotinine tests will be stored securely by the lab conducting the tests in Mumbai, India, for 48 hours after collection.

### Compensation Details

All study participants will receive small monetary incentives to express our appreciation for their participation. Practitioners will receive INR 500 (about US $6) for completing each of the following activities: attending training, registering each patient, completing a 3-month posttraining follow-up survey, and completing a 6-month posttraining follow-up survey. Patients will receive INR 500 (about US $6) when they register for the study, INR 200 (a little over US $2) for completing each of the 1-week, 1-month, and 3-month follow-ups, and INR 500 (about US $6) for completing the 6-month follow-up. The patients who undergo cotinine testing will also receive INR 500 (about US $6) for completing the test. Each of the health and TB NGO sites will receive a one-time payment of INR 10,000 (about US $120) per practitioner participating to account for overhead costs. Given that the dentist practitioners operate their own practices, they will not receive a site-level incentive. These amounts were selected based on the NSF team’s past experiences delivering similar programs to ensure that the amounts are enough to be meaningful to the study populations but not coercive.

### Additional Considerations

All patients in both intervention and control arms will be provided with a pamphlet on the ill effects of tobacco use. Although this is not a “standard of care” in India at this time, it is a common intervention and also accounts for ethical requirements given the degree to which the patients are marginalized and underserved.

No generative language models have been used in this project or this paper.

### Ethical Considerations

This study was approved by the Harvard Longwood Campus IRB in Boston (IRB19-0562) on April 25, 2019, and the Joint Ethics Committee for Narotam Sekhsaria Foundation and Salaam Bombay Foundation in Mumbai (JEC/NSF-SBF nAl9lOS) on August 5, 2019. It is registered in ClinicalTrials.gov of the US National Institutes of Health and in the Clinical Trials Registry of India.

## Results

Recruitment for the RCT commenced in November 2023. As of July 2024, 102% (135/132) have been recruited and trained, including both the intervention and control arms of all 3 setting types. The 3 extra practitioners trained will provide additional help to address attrition; recruitment and training of practitioners are complete. Approximately 36% of patients (1687/4688) have been registered. The first follow-up has been completed for 93% (1460/1574) of the eligible patients; the second follow-up has been completed for 90% (1089/1204) of the eligible patients; the third follow-up has been completed for 87% (583/667) of the eligible patients; and the fourth follow-up has been completed for 76% (188/248) of the eligible patients.

## Discussion

### Principal Findings

In terms of effectiveness outcomes, it is hypothesized that those patients who participate in the adapted LifeFirst SWASTH program will be more likely to have been abstinent from tobacco for 30 consecutive days at the final (6-month) follow-up compared to those patients in the control arm. Furthermore, the patients assigned to the intervention arm will have decreased their tobacco use, even if they did not quit entirely, compared to those in the control arm at the 6-month follow-up.

There are several limitations to be noted. Although 20% of the subsample will provide biomarker-verified cessation outcomes, the majority of patient-level cessation outcomes will be based on self-report. This allows for the possibility of social desirability bias, as tobacco users may under-report their use. Loss to follow-up, especially in low-resource community settings, remains a limitation that may result in potential bias in missing data for engaging patients for follow-ups and outcome measures. While randomization at the cluster level is an effective approach to mitigate potential contamination, it is possible that even a large cluster RCT may not result in a balance of patient-level characteristics between the 2 arms. Selection bias at both patient- and health care provider or practice levels could affect the causal rigor in ascertaining the trial effect. This trial aims to recruit a large sample of current tobacco users across multiple low-resource clinical settings. The large samples of both health care providers and patients yield important information about the delivery and receipt of the intervention, which will eventually affect tobacco cessation. The longitudinal design involving both short- and long-term follow-ups and repeated measures of tobacco cessation outcomes including biomarker verification on a subset of patients will advance our understanding of both proximal and distal effects of this trial. Longitudinal analysis of these repeated-measures outcomes will elucidate the impact of this pragmatic cessation trial on the behavioral trajectories of tobacco use severity and cessation outcomes.

### Conclusions

Given the low-resource and high-reach nature of the intervention, LifeFirst SWASTH could move the needle on tobacco cessation among marginalized groups. Simple brief advice programs have been shown to have an impact of 1%-3% increased cessation rates over no advice, which could translate to 7.8-23.4 million fewer tobacco users in India [45,46]. Programs such as LifeFirst SWASTH that incorporate further counseling are expected to be even more impactful.

In 2022, for the first time, nicotine replacement medications were added to the Indian Ministry of Health and Family Welfare’s List of Essential Medicines, marking an important step in the effort to address tobacco use in India. Ultimately, this will allow insurance to cover the medications, and generally, access will increase [10,47]. However, some medications, such as additional pharmacotherapies (bupropion, varenicline, and others) that would benefit smokeless tobacco users, were not included in the list [10]. Brief advice and counseling interventions such as LifeFirst SWASTH do address smokeless tobacco use and will pave the way for interventions to include both behavioral and pharmacotherapeutic components by preparing the system to support both in tandem.

Furthermore, this study has the potential to promote health equity. It is a free intervention that is primarily delivered over the phone, minimizing the burden for lower-SES patients. It will advance the implementation science literature by focusing exclusively on lower-SES populations and identifying ways to tailor implementation strategies to meet their unique needs [48].

LifeFirst SWASTH, if found to be effective in terms of cessation outcomes and implementation processes, has the potential to be scaled to other settings in India and other LMICs. The study will be conducted in low-resource settings that will reach many patients, which will increase the impact if demonstrated to be effective and then scaled. It will use task-shifting and a mobile app that can be tailored to different settings, also enabling scalability. Additionally, its findings will build the literature for translating EBIs from high-income countries to LMICs and from high- to low-resource settings.

## Abbreviations

5A’s framework: ask, advise, assess, assist, and arrange framework
CHW: community health worker
EBI: evidence-based intervention
GATS-2: 2016-2017 Global Adult Tobacco Survey
IRB: institutional review board
LifeFirst SWASTH: LifeFirst Supporting Wellbeing among Adults by Stopping Tobacco Habit
LMIC: low- and middle-income country
MMR: Mumbai Metropolitan Region
NGO: nongovernmental organization
NSF: Narotam Sekhsaria Foundation
PPA: point prevalence abstinence
RCT: randomized controlled trial
SES: socioeconomic status
TB: tuberculosis
WHO: World Health Organization
